# Supplementary material for: Assessment of Diabetes-Related Knowledge and Dietary Patterns Among Type 2 Diabetes Mellitus Patients in Central Saudi Arabia: Insights for Tailored Health Education Strategies
Source: Healthcare (Basel). 2025 Jan 24;13(3):233. doi: 10.3390/healthcare13030233 (PMC11818046; doi:10.3390/healthcare13030233)
Supplement: Supplementary file 1 [file healthcare-13-00233-s001.zip › healthcare-3408166-supplementary.pdf]

**Part 1: Patient Demographic characteristics information:**		
1	Gender	□ Male<br>□ Female
2	Age (Specify in years)	
3	Marital status	□ Single<br>□ Married<br>□ Divorces<br>□ Widow
4	Educational level	□ Up to Primary<br>□ Secondary<br>□ Higher education (University)
5	Occupation status	□ Employee in government<br>□ Employee in private<br>□ Unemployed (including students, housewives)<br>□ Retired
6	Monthly income	□ Less than 5000 SAR<br>□ 5000-10000 SAR<br>□ More than 10000 SAR
7	Is there any family member with diabetes	□ Yes<br>□ No
8	How many years do you suffer with diabetes?	Specify the duration
9	How do you control your diabetes?	Tablets<br>Insulin<br>Both insulin and Tablets
10	Do you follow up at health centers as per the advised schedule?	No<br>Yes
11	Do you suffer from any other chronic diseases such as kidney problem, heart disease, hypertension, etc	No<br>Yes

**Part 2: Level of Knowledge of diabetes and dietary control**				
content		Yes	No	I don’t know
1	Consuming more sugar and other sweet dishes is a cause of diabetes.			
2	If I am diabetic, my kids have a higher chance of developing diabetic.			
3	Diabetes can be cured*			
4	A fasting blood sugar level of 200 is too high.			
5	Diabetes can damage my kidneys.			
6	Glycosylated hemoglobin (HbA1c) is a test that blood glucose level in the past week.*			
7	Unsweetened fruit juice raises blood glucose levels			
8	A can of diet soft drink can be used for treating low blood glucose levels.*			
9	Eating foods with high calories such as carbohydrate rich food increase blood glucose level*			
10	|Eating foods lower in fat can decrease the risk of diabetes related complications such as heart disease.			

* Reverse scoring

Part 3: Level of Dietary Patterns of Patient of Dietary Control						
content		Never	Rarely (once a week or less)	Occasionally (2-3 times a week)	Often (4-6 times a week)	Daily
1	How often do you consume sugary beverages such as soda, fruit juices, or sweetened coffee/tea per week?					
2	How frequently do you include whole grains in your diet (e.g., whole wheat bread, brown rice)?					
3	How often do you consume processed or fast foods (e.g., burgers, fries, pizza) in a week?					
4	How often do you consume fatty or fried foods (e.g., fried chicken, potato chips)?					
5	How often do you consume lean sources of protein (e.g., poultry, fish, legumes) in a week?					
6	How often do you consume sweets between meals?					
7	How often do you consume healthy snacks between meals (e.g., nuts, yogurt, healthy snacks)?					
8	How often do you consume low-fat or fat-free dairy products?					
9	How often do you consume non-starchy vegetables (e.g., leafy greens, broccoli)?					
10	How often do you eat fruits that are low in sugar, like apples or berries?					
